# Effect of HIV on the Frequency and Number of *Mycobacterium tuberculosis–*Specific CD4^+^ T Cells in Blood and Airways During Latent *M. tuberculosis* Infection

**DOI:** 10.1093/infdis/jix529

**Published:** 2017-10-05

**Authors:** Rubina Bunjun, Catherine Riou, Andreia P Soares, Narjis Thawer, Tracey L Müller, Agano Kiravu, Zekarias Ginbot, Tolu Oni, Rene Goliath, Barbara Kalsdorf, Florian von Groote-Bidlingmaier, Willem Hanekom, Gerhard Walzl, Robert J Wilkinson, Wendy A Burgers

**Affiliations:** 1 Institute of Infectious Disease and Molecular Medicine, University of Cape Town, Cape Town, South Africa; 2 Division of Medical Virology, Department of Pathology, University of Cape Town, Cape Town, South Africa; 3 Wellcome Centre for Infectious Diseases Research in Africa, University of Cape Town, Cape Town, South Africa; 4 Division of Public Health Medicine, School of Public Health and Family Medicine, University of Cape Town, Cape Town, South Africa; 5 Division of Pulmonology, South Africa Department of Science and Technology–National Research Foundation, Cape Town, South Africa; 6 Centre of Excellence for Biomedical Tuberculosis Research, South Africa Department of Science and Technology–National Research Foundation, Cape Town, South Africa; 7 Division of Molecular Biology and Human Genetics, Faculty of Medicine and Health Sciences, Stellenbosch University, Cape Town, South Africa; 8 South African Medical Research Council Centre for Tuberculosis Research, Cape Town, South Africa; 9 Division of Clinical Infectious Diseases, Research Center Borstel, Germany; 10 Francis Crick Institute, London, United Kingdom; 11 Department of Medicine, Imperial College London, London, United Kingdom

**Keywords:** Human immunodeficiency virus, *Mycobacterium tuberculosis*, coinfection, lung, bronchoalveolar lavage, CD4^+^ T-cell responses, adaptive immunity

## Abstract

Human immunodeficiency virus type 1 (HIV) infection substantially increases the risk of developing tuberculosis. There is extensive depletion of *Mycobacterium tuberculosis*–specific CD4^+^ T cells in blood during early HIV infection, but little is known about responses in the lungs at this stage. Given that mucosal organs are a principal target for HIV-mediated CD4^+^ T-cell destruction, we investigated *M. tuberculosis*–specific responses in bronchoalveolar lavage (BAL) from persons with latent *M. tuberculosis* infection and untreated HIV coinfection with preserved CD4^+^ T-cell counts. *M. tuberculosis–*specific CD4^+^ T-cell cytokine (interferon γ, tumor necrosis factor α, and interleukin 2) responses were discordant in frequency and function between BAL and blood. Responses in BAL were 15-fold lower in HIV-infected persons as compared to uninfected persons (*P* = .048), whereas blood responses were 2-fold lower (*P* = .006). However, an increase in T cells in the airways in HIV-infected persons resulted in the overall number of *M. tuberculosis–*specific CD4^+^ T cells in BAL being similar. Our study highlights the important insights gained from studying *M. tuberculosis* immunity at the site of disease during HIV infection.

## Teaser

Human immunodeficiency virus infection leads to lower frequencies of CD4^+^ T cells specific for *Mycobacterium tuberculosis* in blood and lungs. However, an influx of T cells to the lungs results in similar absolute numbers of specific CD4^+^ T cells, compared with uninfected individuals.

Tuberculosis is one of the leading causes of infectious disease globally, with >10 million cases and 1.8 million deaths in 2015 [1]. Of those who developed tuberculosis, 12% were infected with human immunodeficiency virus (HIV), and tuberculosis was the cause of over one third of deaths among HIV-infected persons. This is despite the scale-up of antiretroviral therapy (ART) and the increasing provision of isoniazid therapy to prevent tuberculosis in HIV-infected persons [2].

Whilst the majority of opportunistic infections develop during advanced HIV-associated immunodeficiency, tuberculosis occurs over a wide range of CD4^+^ T-cell counts [2–4]. The risk of developing tuberculosis has been reported to double within the first year after HIV infection [5] and increases with immunodeficiency, and it is up to 26-fold greater among individuals with late untreated infection, compared with HIV-uninfected individuals [1, 3, 6].

The majority of studies of *Mycobacterium tuberculosis* immunity in HIV–*M. tuberculosis* coinfection focus on characterizing T-cell responses to *M. tuberculosis* in blood. Both quantitative and qualitative defects in the CD4^+^ T-cell response to *M. tuberculosis* have been reported in HIV-infected persons with latent *M. tuberculosis* infection [7–11]. Lung responses to *M. tuberculosis* and depletion of CD4^+^ T cells in the airways are considerably less well characterized during HIV infection. HIV has been described as a disease of the mucosal immune system, owing to profound and early CD4^+^ T-cell depletion at mucosal sites such as the gastrointestinal tract [12, 13]. The majority of CD4^+^ T cells in the lungs, sampled by bronchoalveolar lavage (BAL), are CCR5^+^ memory cells [14, 15], the primary target for HIV infection. Despite HIV RNA being readily detectable in BAL fluid [16–19], the frequency of CCR5^+^ CD4^+^ T cells has been reported to be relatively maintained in BAL during HIV infection [14, 20].

*M. tuberculosis–*specific CD4^+^ T-cell responses in the lungs in HIV infection have been examined in only a few studies. A lower frequency of BAL CD4^+^ T-cell responses in immunosuppressed HIV-infected individuals as compared to HIV-uninfected individuals has been reported, and the frequency and function of these cells is distinct from those in blood [15, 17, 21]. Since *M. tuberculosis–*specific CD4^+^ T cells are preferentially and substantially depleted in blood early in HIV infection [8, 22], we sought to investigate *M. tuberculosis* responses and CD4^+^ T-cell depletion in the airways prior to substantial immunodeficiency and their relationship with *M. tuberculosis–*specific responses in blood. We characterized the frequency and absolute number of T cells, the HIV load, and *M. tuberculosis–*specific T-cell responses in BAL and blood specimens from moderately immunosuppressed and untreated HIV-infected and HIV-uninfected individuals with latent *M. tuberculosis* infection.

## METHODS

### Study Participants

Participants were recruited from Cape Town, South Africa, into 2 groups: 25 ART-naive HIV-seropositive persons with CD4^+^ T-cell counts of >400 cells/mm^3^ (median age, 31 years; 96% female) and 25 HIV-seronegative persons (median age, 23 years; 60% female). HIV RNA levels were determined using an Abbott m2000 RealTime HIV-1 assay, and blood CD4^+^ T-cell counts were determined by the Flow-CARE PLG CD4 test. All volunteers were sensitized to *M. tuberculosis*, based on positive results of an interferon γ (IFN-γ) release assay (IGRA; Quantiferon, Cellestis), and tuberculosis was excluded on the basis of symptoms, radiological evidence, and BAL fluid culture results. Previous history of tuberculosis was excluded in all participants except for 2, in whom tuberculosis had occurred >10 years prior to enrollment. This study was approved by the Research Ethics Committees of the University of Cape Town (REF158/2010) and Stellenbosch University (N10/08/275). All participants provided written, informed consent.

### Collection and Processing of Blood Specimens and BAL Fluid

Blood specimens were collected and processed within 4 hours. Flexible bronchoscopy was performed while participants were conscious and sedated. Saline (160 mL) was instilled into the right middle lobe in 20-mL aliquots, aspirated, and stored on ice until processing. BAL samples were centrifuged, and the cell pellet was washed with cold phosphate-buffered saline (Sigma) and filtered through a 100-μm cell strainer (CellTrics, Partec). Acellular BAL fluid was stored at −80°C, and cells underwent immunological analysis while fresh. To correct for epithelial lining fluid (ELF) dilution due to variable fluid volumes recovered (median, 78 mL; interquartile range [IQR], 66–93 mL), the urea method was used (QuantiChrom, Clonagen) as described elsewhere [23]. BAL viral loads and BAL cell counts were standardized according to the volume of ELF sampled (median, 1 mL; IQR, 0.75–1.64 mL) and are expressed as the number of cells per milliliter of ELF.

BAL cells were counted using Trypan Blue exclusion and differentially stained (RapidDiff, Clinical Sciences Diagnostics). A median of 10.6 × 10^6^ cells per BAL specimen (IQR, 6.9× 10^6^–17.6 × 10^6^), or 8.7 × 10^6^ cells/mL ELF, were obtained (Supplementary Table 1). The absolute number of T lymphocytes in BAL fluid was calculated using the frequencies of live CD3^+^, CD4^+^, or CD8^+^ T cells from an ex vivo flow cytometry–based phenotyping panel (for specimens from 38 participants) and by microscopy.

### In Vitro Stimulation of Blood and BAL Cells

Whole-blood stimulation was performed as previously described [24]. Briefly, heparinized whole blood was incubated at 37°C for 12 hours with purified protein derivative (PPD) of *M. tuberculosis* (20 μg/mL) or phorbol 12-myristate 13-acetate (0.01 μg/mL) and ionomycin (1 μg/mL), in the presence of anti-CD28 and anti-CD49d (10 ng/mL and 4 ng/mL, respectively). Unstimulated cells were incubated with costimulatory antibodies only. Brefeldin A (5 μg/mL) was added after 7 hours. After incubation, red blood cells were lysed, and the cell pellet was stained with a violet viability dye, ViViD (Molecular Probes), treated with FACS Lyse (BD), and cryopreserved in 10% dimethyl sulfoxide in fetal calf serum.

Fresh BAL cells underwent similar stimulation in R10 medium (Roswell Park Memorial Institute 1640 medium with 10% fetal calf serum) with the addition of 0.02 mg/mL DNase I, 50 U/mL of penicillin-streptomycin, and 0.8 mg/mL of Fungin. BAL cells were stained with ViViD, treated with FACS Lyse, and stained. BAL cytokine data are reported for 30 of 50 participants (16 with and 14 without HIV infection). The remaining 20 participants had insufficient BAL lymphocyte yields to perform T-cell stimulation assays (<10 × 10^6^ total live BAL cells and/or <2 × 10^5^ total lymphocytes, based on Trypan and differential counts, respectively).

### Intracellular Cytokine Staining and Flow Cytometry

Unstimulated BAL cells were stained ex vivo with anti-CD3-PE-Cy7 and CCR5-PE (both from BD) and with CD4-PE-Cy5.5 and CD8-Qdot705 (both from Invitrogen). Freshly stimulated BAL cells and stimulated cryopreserved blood cells were washed and stained with anti-CD4-PE-Cy5.5 and CD8-Qdot705 (both from BD), permeablized, and stained intracellularly with CD3-APC-H7, IFN-γ-Alexa700, and interleukin 2 (IL-2)-APC (all from BD) and with tumor necrosis factor α (TNF-α)-PE-Cy7 (eBiosciences). Cells were acquired on a BD Fortessa, using FACSDiva software, and data were analyzed using FlowJo (TreeStar) and Pestle and Spice [25]. A positive cytokine response was defined as a level that was twice the background level, a net response of >0.05%, and an event cutoff of 10 events, and all data are reported after subtraction of the background level.

### Statistical Analysis

Statistical analyses were performed using Prism 5 (GraphPad). Nonparametric tests (the Mann-Whitney *U* test, the Wilcoxon matched pairs test, and the Spearman rank test) were used for all comparisons. A *P* value of < .05 was considered statistically significant.

## RESULTS

### Cohort and Clinical Characteristics

Blood and bronchoalveolar lavage (BAL) samples were collected from 25 HIV-infected and 25 HIV-uninfected persons sensitized to *M. tuberculosis*, as evidenced by a positive result of an IFN-γ release assay. The clinical characteristics of the participants are summarized in Table 1. HIV-infected participants were ART naive, had well maintained CD4^+^ T-cell counts (median, 619 cells/mm^3^; IQR, 533–782 cells/mm^3^), and exhibited a wide range of plasma viral loads (median, 6383 RNA copies/mL; IQR, 3548–16449 RNA copies/mL). HIV RNA was detectable in BAL fluid from 20 of 25 participants, with a median load of 10700 RNA copies/mL ELF (IQR, 2780–27287 RNA copies/mL ELF). There was a significant positive correlation between viral load in plasma and BAL (*P* < .0001; r = 0.6958; data not shown), consistent with published studies [16, 18, 19].

**Table 1.** Clinical Characteristics of Study Participants, by Human Immunodeficiency Virus Status

Characteristic	Uninfected (n = 25)	Infected (n = 25)
**Blood CD4** ^**+**^ **T-cell count, cells/mm** ^**3**a^	813 (676–933)	619 (533–782)
**Plasma viral load, RNA copies/mL**	…	6383 (3548–16 449)
**BAL viral load,** **RNA copies/mL ELF**	…	10 700 (2780–27 287)

Data are median values (interquartile ranges).

Abbreviations: BAL, bronchoalveolar lavage; ELF, epithelial lining fluid.

^a^ *P* = .0016.

### Effect of HIV on the Cellular Composition of BAL Fluid

**Figure 1.**
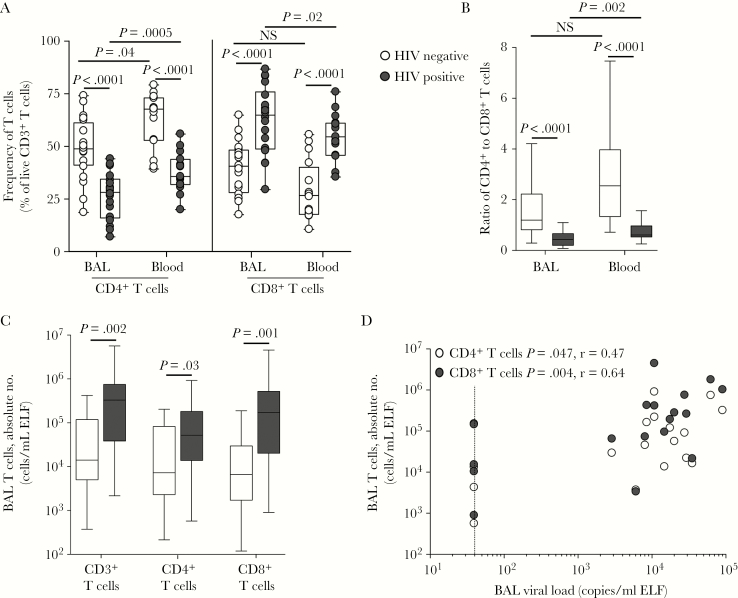
T-cell frequencies and absolute numbers in blood and bronchoalveolar lavage. *A*, The frequencies of CD4^+^ and CD8^+^ T cells in blood and bronchoalveolar lavage (BAL) of human immunodeficiency virus (HIV)–infected (n = 18) and uninfected (n = 20) individuals, for whom ex vivo flow cytometry phenotyping data were available. Data are shown as box and whisker plots, with horizontal bars representing medians, boxes denoting interquartile ranges, and whiskers denoting ranges. Each dot represents an individual. The frequency of CD4^+^ and CD8^+^ T cells is shown as a percentage of live CD3^+^ T lymphocytes, determined by flow cytometry. *B*, Ratios of CD4^+^ to CD8^+^ T cells in BAL and blood specimens for HIV-infected (black bars) and uninfected (white bars) individuals. *C*, The absolute number of CD3^+^, CD4^+^, and CD8^+^ T cells in BAL from HIV-infected and uninfected individuals. *D*, The association between absolute CD4^+^ and CD8^+^ T-cell counts and BAL viral load. The dotted line indicates the limit of detection of the viral load assay. Statistical comparisons were performed using the nonparametric Mann-Whitney test for unpaired comparisons, the Wilcoxon test for paired samples, and Spearman rank correlation. ELF, epithelial lining fluid; NS, not significant.

The cellular content of BAL consisted primarily of alveolar macrophages (>90%) and smaller populations of lymphocytes and neutrophils (Supplementary Table 1). Compared with HIV-uninfected individuals, HIV-infected individuals had a significantly lower proportion of macrophages (median, 96% [IQR, 92.1%–97%] vs 92.8% [IQR, 80.7%–96.1%]; *P* = .031) and a higher proportion of lymphocytes (median, 3% [IQR, 1.9%–6%] vs 6.2% [IQR, 3.5%–16.8%], respectively; *P* = .005). As expected, HIV-infected participants had significantly lower frequencies of CD4^+^ T cells in BAL and blood specimens, compared with HIV-uninfected participants (*P* < .0001 for both comparisons; Figure 1A), and significantly higher frequencies of CD8^+^ T cells in BAL and blood specimens (*P* < .0001 for both comparisons). In HIV-uninfected persons, the ratio of CD4^+^ to CD8^+^ T cells in BAL fluid was lower than in blood specimens (median, 1.2 [IQR, 0.82–2.22] and 2.55 [IQR, 1.34–3.97]; Figure 1B). As expected, HIV infection led to a skewed ratio in blood specimens (median, 0.61; IQR, 0.53–0.97), and for BAL fluid the ratio was 0.44 (IQR, 0.2–0.66). Since these data reflect only the distribution of cells but not the extent of their depletion or expansion, we calculated absolute numbers of CD3^+^, CD4^+^ and CD8^+^ T cells in BAL fluid (Figure 1C). HIV-infected individuals had significantly greater absolute numbers of CD3^+^ lymphocytes than HIV-uninfected individuals (median, 329243 cells/mL ELF vs 14085 cells/mL ELF; *P* = .002), consisting of 26-fold more CD8^+^ T cells (*P* = .001) and 7-fold more CD4^+^ T cells (*P* = .03). When we examined CCR5 expression on BAL CD4^+^ T cells, a median of 76% expressed CCR5, regardless of HIV status (data not shown), implying that these cells are potential targets for HIV, despite their higher absolute number in BAL fluid as compared to the value for HIV-uninfected individuals. Absolute numbers of BAL CD4^+^ T cells (*P* = .047; r = 0.47) and CD8^+^ T cells (*P* = .004; r = 0.64) correlated positively with BAL viral load (Figure 1D). In contrast, blood specimens exhibited the expected inverse correlation between plasma viral load and absolute CD4^+^ T-cell count (*P* = .0005; r = −0.64; data not shown).

Overall, these data show that despite the moderate depletion of CD4^+^ T cells in blood specimens from HIV-infected participants, they had a greater number of CD4^+^ T cells in BAL fluid as compared to findings for uninfected participants.

### Lower Frequencies but Preserved Absolute *M. tuberculosis–*Specific CD4^+^ T-Cell Counts in BAL Fluid During HIV Infection

**Figure 2.**
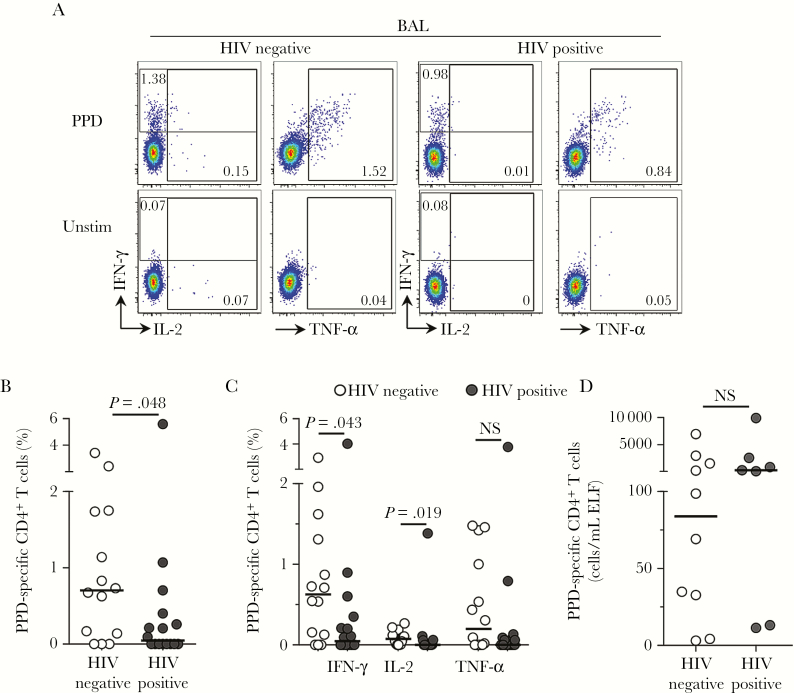
Bronchoalveolar CD4^+^ T-cell responses to *Mycobacterium tuberculosis* in human immunodeficiency virus (HIV)–infected and uninfected individuals. *A*, Representative flow cytometry plots of interferon γ (IFN-γ), interleukin 2 (IL-2), and tumor necrosis factor α (TNF-α) production by CD4^+^ T cells after stimulation with *M. tuberculosis* purified protein derivative (PPD) in 1 HIV-infected and 1 HIV-uninfected study participant. The frequency of cytokine-producing cells is shown as a percentage of the total CD4^+^ T-cell population, after gating on live, CD3^+^ T lymphocytes. Unstim, unstimulated control. *B*, The total frequency of CD4^+^ T cells producing any cytokine in response to *M. tuberculosis* PPD in HIV-infected (n = 16) and HIV-uninfected (n = 14) individuals. Twenty participants had insufficient lymphocyte yields from BAL fluid to perform T-cell stimulation assays. *C*, Individual cytokine responses to PPD in HIV-infected and uninfected individuals. *D*, The absolute number of PPD-specific CD4^+^ T cells, calculated by adjusting the frequency of cells for the estimated BAL CD4^+^ T-cell count in HIV-infected and HIV-uninfected individuals for whom the estimated absolute CD4^+^ T-cell count in BAL fluid was determined. Only responders are plotted on the graph. Each dot represents 1 individual, and horizontal bars represent the median. Open circles represent HIV-uninfected individuals, and filled circles represent HIV-infected individuals. Statistical comparisons were performed using the nonparametric Mann-Whitney test. ELF, epithelial lining fluid; NS, not significant.

We next examined the effect of HIV on *M. tuberculosis–*specific CD4^+^ T-cell responses in the airways. Owing to the low number of lymphocytes present in BAL, 30 participants had a sufficient number of cells available to perform the stimulation assay. Figure 2A shows representative flow cytometry plots of IFN-γ, IL-2, and TNF-α CD4^+^ T-cell responses to *M. tuberculosis* PPD. We detected responses in 79% of HIV-uninfected individuals (11 of 14) and 50% of HIV-infected individuals (8 of 16; Figure 2B). The frequency of PPD-specific CD4^+^ T cells producing any cytokine was significantly lower (15-fold) in HIV-infected participants as compared to uninfected individuals (median, 0.05% [IQR, 0%–0.37%] and 0.7% [IQR, 0.1%–1.74%]; *P* = .048; Figure 2B). When considering only individuals with a detectable response in BAL fluid, median frequencies for HIV-infected participants and uninfected participants were 0.33% and 0.83%, respectively, equating to a 2.5-fold lower median frequency in the former group. Upon examining cytokines individually, HIV-infected individuals had significantly lower frequencies of CD4^+^ T cells producing IFN-γ (*P* = .043) or IL-2 (*P* = .019), compared with HIV-uninfected participants (Figure 2C). Since we found that higher numbers of T cells were present in the lung during HIV infection (Figure 1C), we adjusted the frequencies of *M. tuberculosis–*specific CD4^+^ T cells for CD4^+^ T-cell counts in BAL fluid. Consequently, we found that in those with a detectable response in BAL fluid, there was no significant difference in the absolute number of BAL CD4^+^ T cells responding to *M. tuberculosis* between HIV-infected and HIV-uninfected participants (Figure 2D). Thus, our data show that half of HIV-infected IGRA-positive individuals had no detectable *M. tuberculosis–*specific CD4^+^ T-cell responses in BAL. In those with detectable BAL responses, there was a similar absolute number of *M. tuberculosis–*specific CD4^+^ T cells in BAL fluid, when adjusted for the increased T-cell numbers in BAL fluid during HIV infection.

### Lower Frequencies of *M. tuberculosis–*Specific CD4^+^ T Cells in Blood in HIV-Infected Individuals Despite Well-Preserved CD4^+^ T-Cell Counts

**Figure 3.**
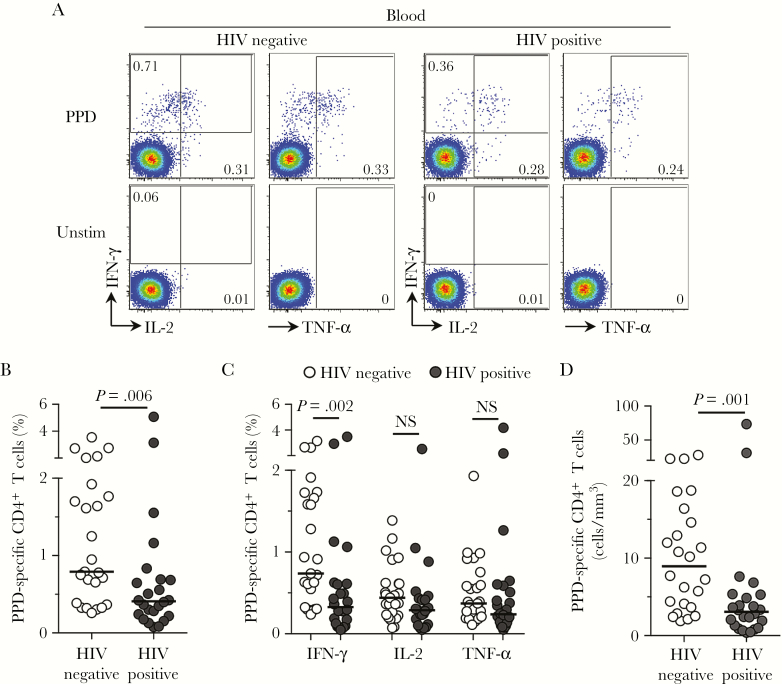
Blood CD4^+^ T-cell responses to *Mycobacterium tuberculosis* in human immunodeficiency virus (HIV)–infected and uninfected individuals. *A*, Representative flow cytometry plots of the production of interferon γ (IFN-γ), interleukin 2 (IL-2), and tumor necrosis factor α (TNF-α) by CD4^+^ T cells after stimulation with *M. tuberculosis* purified protein derivative (PPD) in 1 HIV-infected and 1 HIV-uninfected study participant. The frequency of cytokine-producing cells is shown as a percentage of the total CD4^+^ T-cell population after gating on live, CD3^+^ T lymphocytes. Unstim, unstimulated control. *B*, The total frequency of CD4^+^ T cells producing any cytokine in response to PPD in HIV-infected (n = 25) and HIV-uninfected (n = 25) individuals. *D*, The frequency of individual cytokine responses to PPD in HIV-infected and HIV-uninfected individuals. *E*, The absolute number of PPD-specific CD4^+^ T cells, calculated by adjusting the frequency of cells for the CD4^+^ T-cell count in HIV-infected and uninfected participants. The CD4^+^ T-cell count was unavailable for 1 HIV-uninfected participant. Each dot represents 1 individual, and horizontal bars represent the median. Open circles represent HIV-uninfected individuals, and filled circles represent HIV-infected individuals. Statistical comparisons were performed using the nonparametric Mann-Whitney test.

We next analyzed *M. tuberculosis–*specific CD4^+^ T-cell responses in peripheral blood specimens from the same individuals. Previous reports suggest that depletion of *M. tuberculosis–*specific CD4^+^ T cells in blood occurs early after HIV infection [8, 22]. Although the duration of HIV infection for our study participants was not known, their well-maintained CD4^+^ T-cell counts in the absence of ART (median, 619 cells/mm^3^) likely reflected relatively early infection. A representative example of CD4^+^ T-cell cytokine production in blood in response to PPD is depicted in Figure 3A. We examined adaptive immunity in blood specimens, and all 50 participants had a detectable PPD response, which correlated significantly with the QFT response (*P* = .0067; r = 0.38; data not shown). Similar to our observations in BAL fluid, the total frequency of *M. tuberculosis–*specific CD4^+^ T cells in blood specimens was significantly lower (by 2-fold) in HIV-infected individuals as compared to HIV-uninfected subjects (median, 0.41% [IQR, 0.38%–1.85%] and 0.79% [IQR, 0.23%–0.69%]; *P* = .006; Figure 3B). Analysis of each cytokine (Figure 3C) revealed that HIV-infected individuals had lower frequencies of cells producing IFN-γ, compared with HIV-uninfected individuals (*P* = .002). After adjustment for absolute CD4^+^ T-cell count in blood, this was more pronounced, with 3-fold lower numbers of *M. tuberculosis–*specific cells (*P* = .0012; Figure 3D). In blood specimens, there was a significant positive correlation between frequencies of PPD-specific CD4^+^ T cells and CD4^+^ T-cell counts (*P* = .03; r = 0.45; Supplementary Figure 1*A*), suggesting that the decrease in *M. tuberculosis* CD4^+^ T-cell responses was related to overall CD4^+^ T-cell depletion. We found no relationship between blood *M. tuberculosis* response frequencies and plasma viral load (*P* = .09; r = 0.35; Supplementary Figure 1*B*), or between BAL responses to *M. tuberculosis* and either BAL CD4^+^ T-cell count or BAL viral load (Supplementary Figure 1*C* and 1*D*).

Overall, our data show that even in HIV-infected individuals with well-maintained peripheral CD4^+^ T-cell counts in the absence of ART, the frequency of peripheral *M. tuberculosis–*specific CD4^+^ T cells was significantly lower than in HIV-uninfected subjects.

### Compartmentalization of *M. tuberculosis–*Specific Responses in Blood and BAL Specimens

**Figure 4.**
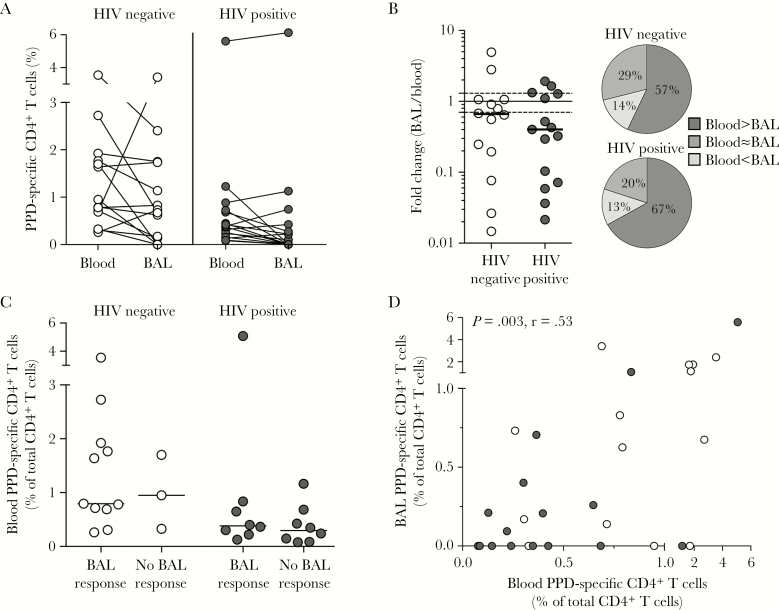
Comparison of blood and bronchoalveolar lavage (BAL) CD4^+^ T-cell responses to *Mycobacterium tuberculosis* in human immunodeficiency virus (HIV)–infected and uninfected individuals. *A*, Comparison of the frequency of *M. tuberculosis* purified protein derivative (PPD)–specific CD4^+^ T cells in blood and BAL specimens from HIV-uninfected (n = 14) and HIV-infected (n = 16) participants. *B*, Fold change in frequencies between blood and BAL specimens in HIV-uninfected and HIV-infected individuals. The dotted line indicates no difference between BAL and blood specimens (fold change of 1) and the solid lines indicate the empirical cutoff of 30% that was used to determine whether responses were different between compartments. Pie charts show the percentage of individuals for whom blood and BAL responses to PPD were different or similar. *C*, The frequency of PPD-specific CD4^+^ T cells in blood specimens from HIV-uninfected and HIV-infected participants with detectable or undetectable BAL responses. *D*, The relationship between CD4^+^ T-cell responses to PPD in blood and BAL specimens in HIV-infected and uninfected individuals. Each dot represents an individual. Statistical analyses were performed using the Wilcoxon test, the Mann-Whitney test, and the Spearman rank test. Unless otherwise indicated, all *P* values were nonsignificant.

*M. tuberculosis* responses in blood are frequently measured in vaccine trials, but whether they are a surrogate for responses in the airways is not clear. Thus, we directly compared the frequencies of *M. tuberculosis–*specific CD4^+^ T-cell responses between blood and BAL specimens in the subgroup of 30 participants from whom we had data for both compartments. There was no difference in the median frequency of *M. tuberculosis* responses between BAL and blood specimens, regardless of HIV infection status (Figure 4A). We then examined the differences between compartments in more detail by calculating the fold change of the frequency of PPD responses between blood and BAL specimens for each participant. We found no difference in the fold change between HIV-uninfected and HIV-infected individuals (Figure 4B). Furthermore, concordant blood and BAL T-cell response frequencies were present in 29% and 20% of HIV-uninfected and HIV-infected participants, respectively (Figure 4B), while the majority had a higher frequency of responses in blood specimens as compared to BAL fluid (57% and 67%, respectively). The proportion of participants with higher T-cell frequencies in BAL fluid than blood specimens was <15%. Individuals with undetectable *M. tuberculosis–*specific responses in BAL fluid had similar blood CD4^+^ T-cell frequencies as those with detectable BAL responses (Figure 4C). When we examined the association between the 2 compartments, we nonetheless observed a direct correlation between the frequency of CD4^+^ T cells specific for PPD in blood and BAL (*P* = .003; r = 0.53; Figure 4D).

In summary, there was an association in response frequencies between the 2 compartments, regardless of HIV infection status. Nevertheless, blood responses did not predict the absence of *M. tuberculosis–*specific BAL responses in one third of the participants.

### Functional Capacity of *M. tuberculosis–*Specific CD4^+^ T Cells Differs Between BAL and Blood Specimens

**Figure 5.**
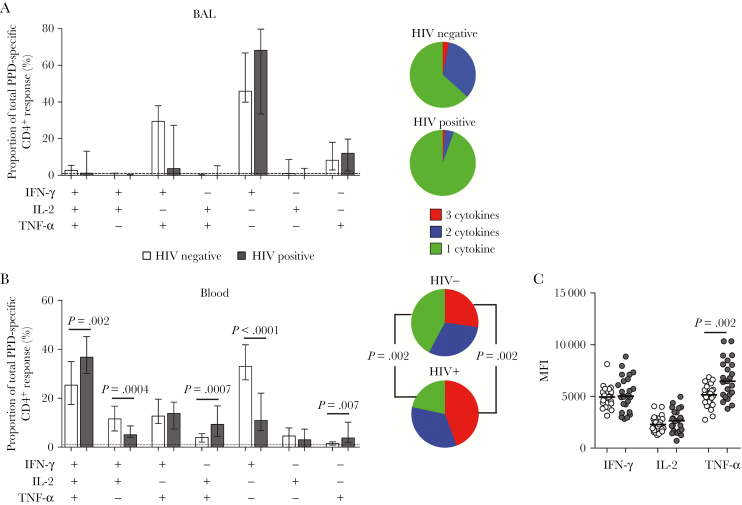
Function of *Mycobacterium tuberculosis* purified protein derivative (PPD) responses in bronchoalveolar lavage (BAL) and blood specimens from human immunodeficiency virus (HIV)–infected and uninfected individuals. *A* and *B*, Populations of CD4^+^ T cells from HIV-infected or uninfected individuals that produced different combinations of the 3 cytokines tested in response to *M. tuberculosis* PPD in BAL (*A*) and blood (*B*) specimens. Open bars represent HIV-uninfected participants, and filled bars represent HIV-infected participants. The tops of the bars depict the median, and the interquartile range is indicated. Pie charts representing the polyfunctional capacity of CD4^+^ T cells responding to PPD are shown next to the bar graphs. The grey slices of the chart represent CD4^+^ T cells producing all 3 cytokines tested, the dark grey slices are cells producing any 2 cytokines in combination, and the light grey slices represent cells producing only 1 cytokine, expressed as a proportion of the total response. *C*, Median fluorescent intensity (MFI) of cytokine production by CD4^+^ T cells responding to *M. tuberculosis* PPD in HIV-infected and uninfected individuals in blood. Only positive cytokine responses were included in the analysis. Each dot represents an individual. Open circles represent HIV-uninfected individuals, and filled circles represent HIV-infected individuals. Statistical comparisons were performed using the Mann-Whitney test. IFN-γ, interferon γ; IL-2, interleukin 2; TNF-α, tumor necrosis factor α.

Finally, we investigated the functional quality of CD4^+^ T-cell responses to *M. tuberculosis* during HIV infection. Cytokine coexpression profiles differed substantially between BAL and blood specimens (Figure 5A and 5B). In BAL fluid, monofunctional IFN-γ–producing CD4^+^ T cells dominated the response to PPD in both HIV-infected and uninfected individuals (median, 46% and 68%, respectively; Figure 5A). The remainder of the cells produced TNF-α alone or in combination with IFN-γ, with negligible polyfunctional responses. There were no significant differences in CD4^+^ T-cell functional profiles between HIV-infected and uninfected individuals. In contrast to BAL fluid, PPD-specific cells from blood specimens exhibited a greater diversity in their functional profiles, primarily as a result of higher IL-2 responses, and were highly polyfunctional (Figure 5B). Moreover, the distribution of functional subsets in blood specimens was significantly different between HIV-uninfected and HIV-infected individuals, with the proportion of cells producing only IFN-γ, as well as the subset producing IFN-γ and IL-2 only, significantly lower in HIV-infected individuals, compared with HIV-uninfected individuals (*P* < .0001 and *P* = .0004, respectively). Furthermore, HIV-infected individuals had a significantly higher proportion of cells expressing IFN-γ, IL-2, and TNF-α; IL-2 and TNF-α only; and TNF-α only (*P* = .002, *P* = .0007 and *P* = .007, respectively), compared with HIV-uninfected individuals. We also noted a significant difference in the median fluorescent intensity of TNF-α in *M. tuberculosis–*specific CD4^+^ T cells, with a higher value in HIV-infected individuals as compared to uninfected individuals (*P* = .002; Figure 5C), whereas no such difference was observed in BAL fluid (data not shown). Of note, while the sex distribution differed between the HIV-infected and uninfected groups, no significant differences in the functional profile or magnitude of PPD-specific CD4^+^ T cells were observed between male and female participants (data not shown).

Taken together, these results suggest specific perturbations in cytokine production in *M. tuberculosis–*specific CD4^+^ T cells during HIV infection, and they highlight distinct HIV-induced changes in blood compared to BAL. Collectively, these data emphasize that *M. tuberculosis* responses in blood do not necessarily reflect the quantity or quality of the *M. tuberculosis* immune responses in the airways.

## DISCUSSION

HIV infection is the greatest recognized risk factor for the development of tuberculosis in high-burden settings [26]. The risk of tuberculosis increases up to 30-fold as CD4^+^ T cells are progressively depleted [26, 27]. CD4^+^ T-helper type 1 (Th1) cells play a critical role in immunity to *M. tuberculosis* [28], and depletion of peripheral CD4^+^ T-cell responses to *M. tuberculosis* during HIV infection has been described in many studies [8, 10, 22, 29–31]. In contrast to the numerous studies of *M. tuberculosis* in blood, the effect of HIV on immunity at the site of infection is considerably less well studied. We examined *M. tuberculosis–*specific lung immunity in HIV-infected persons with moderate immunosuppression. We found a decreased frequency of *M. tuberculosis–*specific CD4^+^ Th1 responses in blood specimens from HIV-infected participants as compared to uninfected persons, consistent with published studies of advanced HIV disease [15, 17, 21]. Several novel observations emerged from BAL in our study: (1) although HIV-infected individuals had lower CD4^+^ T-cell frequencies in BAL fluid, they had a 7-fold greater absolute CD4^+^ T-cell count in these specimens, compared with HIV-uninfected individuals; (2) increased CD4^+^ T-cell numbers in BAL fluid were associated with BAL viral load; (3) in 30 participants in whom we measured lung immunity, one third had no detectable *M. tuberculosis–*specific responses in BAL fluid, despite detectable T-cell responses in blood specimens; (4) although HIV-infected participants had significantly lower frequencies of *M. tuberculosis–*specific CD4^+^ T cells, when those with detectable BAL responses were adjusted for CD4^+^ T-cell numbers in BAL fluid, no significant differences were observed from HIV-uninfected participants. These data highlight disparities in the effect of HIV on CD4^+^ T-cell dynamics in the blood and the lungs and underscore the compartmentalization of *M. tuberculosis–*specific responses between the 2 sites.

Adaptive immunity in blood is used as a surrogate for lung responses, emphasizing the importance of understanding differences between blood and BAL T-cell responses. Approximately two thirds of individuals had a higher *M. tuberculosis* response frequency in blood specimens as compared to BAL fluid, regardless of HIV infection. Although there was a weak positive association between blood and BAL response frequencies, undetectable responses in BAL fluid were unrelated to the magnitude of *M. tuberculosis–*specific CD4^+^ T-cell frequencies in blood specimens. The absence of responses to *M. tuberculosis* in BAL fluid was more pronounced in HIV-infected persons, with 50% having no detectable responses. In addition to HIV-associated depletion, several possibilities could account for the absence of BAL responses. We sampled a single site, and *M. tuberculosis* immunity may not be uniform throughout the lung [32]. Furthermore, we studied *M. tuberculosis–*exposed individuals, among whom the spectrum of *M. tuberculosis* history could range from contained latent infection to cleared infection without persisting *M. tuberculosis–*specific memory responses in the lungs [33]. *M. tuberculosis–*specific CD4^+^ T cells were also functionally distinct between the compartments, with a negligible percentage of polyfunctional PPD-specific CD4^+^ T cells within the BAL CD4^+^ T-cell population, compared with approximately 25% in blood. This is in contrast to studies using mitogens and other pathogens, in which highly polyfunctional CD4^+^ T cells were detected in BAL fluid [14, 20], and thus may be specific to the pathogen studied.

This compartmentalization in frequency and function between blood and lung *M. tuberculosis–*specific responses may have important implications for tuberculosis vaccine development. Recent trials of the first novel tuberculosis vaccine, MVA85A, failed to demonstrate protection against *M. tuberculosis* infection or tuberculosis [34, 35] despite eliciting increased CD4^+^ T-cell responses in blood. An effective tuberculosis vaccine may need to induce immunity to *M. tuberculosis* in the airways. Although BAL is expensive to perform and requires specialized personnel, early vaccine trials could investigate local pulmonary responses in a subset of individuals. Additionally, vaccine delivery into the airways may be a promising strategy to induce long-lived pulmonary immunity and increase vaccine efficacy [36–38].

We found increased CD4^+^ T-cell numbers in BAL fluid during HIV infection despite moderate peripheral CD4^+^ T-cell depletion. The marked increase of both CD4^+^ and CD8^+^ T-cell counts in the airways that we observed was reflective of a degree of lymphocytic alveolitis characteristic of HIV infection [39–41]. The relative maintenance of predominantly CCR5-expressing (and, thus, HIV-susceptible) CD4^+^ memory T cells supports the hypothesis that the lung is a mucosal site distinct from the gastrointestinal tract when it comes to the effects of HIV [14] and may be attributed to the presence of protective β-chemokines [14, 16, 20]. We speculate that the decreased frequency of *M. tuberculosis* CD4^+^ T cells may be offset by an influx of CD4^+^ T cells that normalizes the absolute number of *M. tuberculosis–*specific CD4^+^ T cells and thus keeps the tuberculosis risk relatively low, thus resolving the apparent discrepancy between a substantially decreased *M. tuberculosis–*specific CD4^+^ T-cell frequency but only a doubling of tuberculosis risk in early infection [5].

It is worth noting that we restricted our analyses to blood and BAL CD4^+^ Th1 responses, considered the cornerstone of adaptive *M. tuberculosis* immunity. There is, however, a growing body of evidence that IFN-γ–independent mechanisms may also provide protection against tuberculosis [42]. Further studies, particularly at sites of tuberculosis, are needed to investigate the role of additional Th cell lineages, as well as the possible role of unconventional T cells [43], in protective immunity to *M. tuberculosis*. In addition, early defects in innate immunity in both blood and BAL may influence tuberculosis risk. Last, although BAL is a relatively noninvasive technique for studying immunity in the lungs, a major limitation is the low yield of lymphocytes, enabling us to perform functional immune assays in only 60% of our cohort. Thus, further studies will be required to confirm our findings.

In summary, this study showed that antiretroviral-untreated, *M. tuberculosis–*exposed HIV-infected persons with moderately well-preserved CD4^+^ T-cell numbers had a significant decrease in the frequency of CD4^+^ T-cell responses to *M. tuberculosis* in blood and BAL specimens. However, an increased T-cell count in the airways related to the HIV load may have resulted in a similar absolute number of *M. tuberculosis–*specific CD4^+^ T cells in BAL fluid. Our novel findings emphasize the differences between blood and lung responses to *M. tuberculosis* and highlight the need to better understand *M. tuberculosis* responses in the lungs.

## Supplementary Data

Supplementary materials are available at *The Journal of Infectious Diseases* online. Consisting of data provided by the authors to benefit the reader, the posted materials are not copyedited and are the sole responsibility of the authors, so questions or comments should be addressed to the corresponding author.

## Supplementary Material

Table S1

Fig S1

## Notes

***Acknowledgments.*** We thank the study participants, for providing samples and for their time and commitment to the study; the clinical staff at the Ubuntu HIV-TB clinic and the bronchoscopy suite at Tygerberg Academic Hospital, for providing essential support; Mrs Kathryn Norman, for administrative assistance; Maximilian Lücke, for phlebotomy, cataloguing participant radiography findings, and database assistance; Natalie Strickland and Ramla Tanko, for critical review of the manuscript; and Thomas Scriba, for helpful advice.

***Disclaimer.***  The funders had no role in study design, data collection and analysis, decision to publish, or preparation of the manuscript.

W. A. B., R. B., A. P. S., W. H., and R. J. W. conceived and designed the experiments. R. B., A. P. S., N. K. T., T. L. M., A. K., and Z. G. performed the experiments. R. B., A. P. S., C. R., and W. A. B. analyzed the data. T. O., R. G., G. W., F. v. G.-B., B. K., and R. J. W. contributed reagents, materials, and/or analysis tools. R. B., C. R., and W. A. B. wrote the manuscript. All authors approved the final manuscript.

***Financial support.*** This work was supported by the European and Developing Countries Clinical Trials Partnership (grant TA_08_40200_020); the South African Medical Research Council (SAMRC); the South African National Research Foundation (grant 96841 to R. J. W.); the Wellcome Trust (award 089832/Z/09/Z to W. A. B. and awards 203135 and 104803 to R. J. W.); the Carnegie Corporation (PhD fellowship to R. B.); the University of Cape Town (to R. B.); the Canada Africa Prevention Trials Network (to R. B.); the Office of the Director, National Institutes of Health (grant R21AI115977 to C. R.); the German Center for Infection Research (to B. K.); the Francis Crick Institute (Cancer Research UK, Medical Research Council UK, and Wellcome grant FC0010218 to R. J. W.); the European Union (award FP7-Health-F3-2012–305578 to R. J. W.); and the Strategic Health Innovation Partnership, SAMRC (to R. J. W.).

***Potential conflicts of interest.*** All authors: No reported conflicts. All authors have submitted the ICMJE Form for Disclosure of Potential Conflicts of Interest. Conflicts that the editors consider relevant to the content of the manuscript have been disclosed.
