# Supplementary material for: Effect of HIV on the Frequency and Number of Mycobacterium tuberculosis–Specific CD4+ T Cells in Blood and Airways During Latent M. tuberculosis Infection
Source: J Infect Dis. 2017 Oct 5;216(12):1550–60. doi: 10.1093/infdis/jix529 (PMC5815627; doi:10.1093/infdis/jix529)
Supplement: Table S1 [file jix529_suppl_table_s1.docx]

**Supplementary Table 1: Characteristics of Cells obtained from BAL**

|  | **HIV-uninfected (n=25)** | **HIV-infected (n=25)** | ***P***  **value** |
| --- | --- | --- | --- |
| **Cells/ml ELF**^a^ | 8.0 x 10^6^ (4.3-13.7) | 9.8 x 10^6^ (3.2-16.5) | ns |
| **Alveolar macrophages (%)**^a^ | 96.0 (92.1-97.0) | 92.8 (80.7-96.1) | **0.031** |
| **Lymphocytes (%)**^a^ | 3.0 (1.9-6.0) | 6.2 (3.5-16.8) | **0.005** |
| **Neutrophils (%)**^a^ | 1.0 (0.0-2.0) | 1.0 (0.0-2.0) | ns |

^a^Data are presented as median (interquartile range)
