# Supplementary figures and images for: Effect of HIV on the Frequency and Number of Mycobacterium tuberculosis–Specific CD4+ T Cells in Blood and Airways During Latent M. tuberculosis Infection
Source: J Infect Dis. 2017 Oct 5;216(12):1550–60. doi: 10.1093/infdis/jix529 (PMC5815627; doi:10.1093/infdis/jix529)

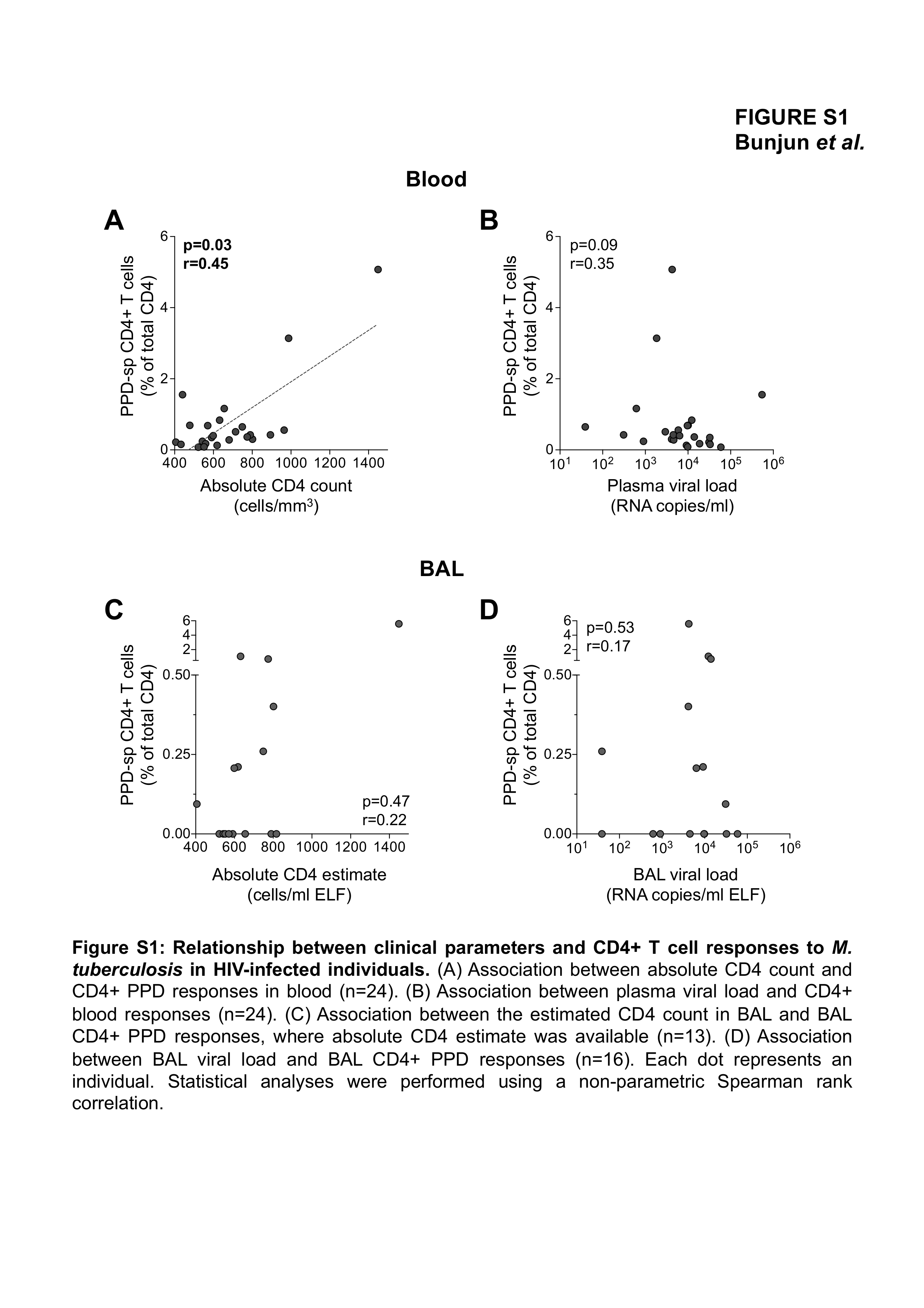

Supplement: Fig S1 [file jix529_suppl_fig_s1.png]
